# Supplementary material for: The combined DNA and RNA synthetic capabilities of archaeal DNA primase facilitate primer hand-off to the replicative DNA polymerase
Source: Nat Commun. 2022 Jan 21;13:433. doi: 10.1038/s41467-022-28093-2 (PMC8782868; doi:10.1038/s41467-022-28093-2)
Supplement: Supplementary file 1 — Supplementary Information [file 41467_2022_28093_MOESM1_ESM.pdf]

Supplementary Information for

The combined DNA and RNA synthetic capabilities of archaeal DNA primase facilitate primer hand-off to the replicative DNA polymerase

Mark D. Greci[1], Joseph D. Dooher[2], and Stephen D. Bell[1,3]

[1]Department of Biology, Indiana University, Simon Hall MSB, 212 S Hawthorne Drive, Bloomington, 47405, USA

[2]Independent scholar

[3]Department of Molecular and Cellular Biochemistry, Indiana University, Simon Hall MSB, 212 S Hawthorne Drive, Bloomington, 47405, USA

* Correspondence to SDB, stedbell@indiana.edu Ph. 812 856 2331

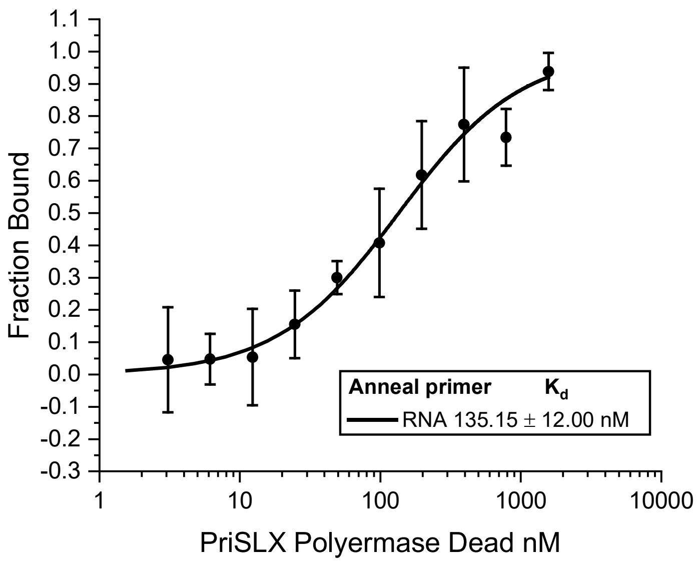

Supplementary Figure 1: Binding analysis of PriSLX Polymerase Dead

K_d determination by fluorescence polarization. Titration series of PriSLX PD (Polymerase Dead) as indicated with 5'-cy5-labeled 19 nt RNA-primer annealed to 80 nt template. 3 independent replicates. Data points are the mean and error bars represent standard deviation. Source data are provided as a source data file.

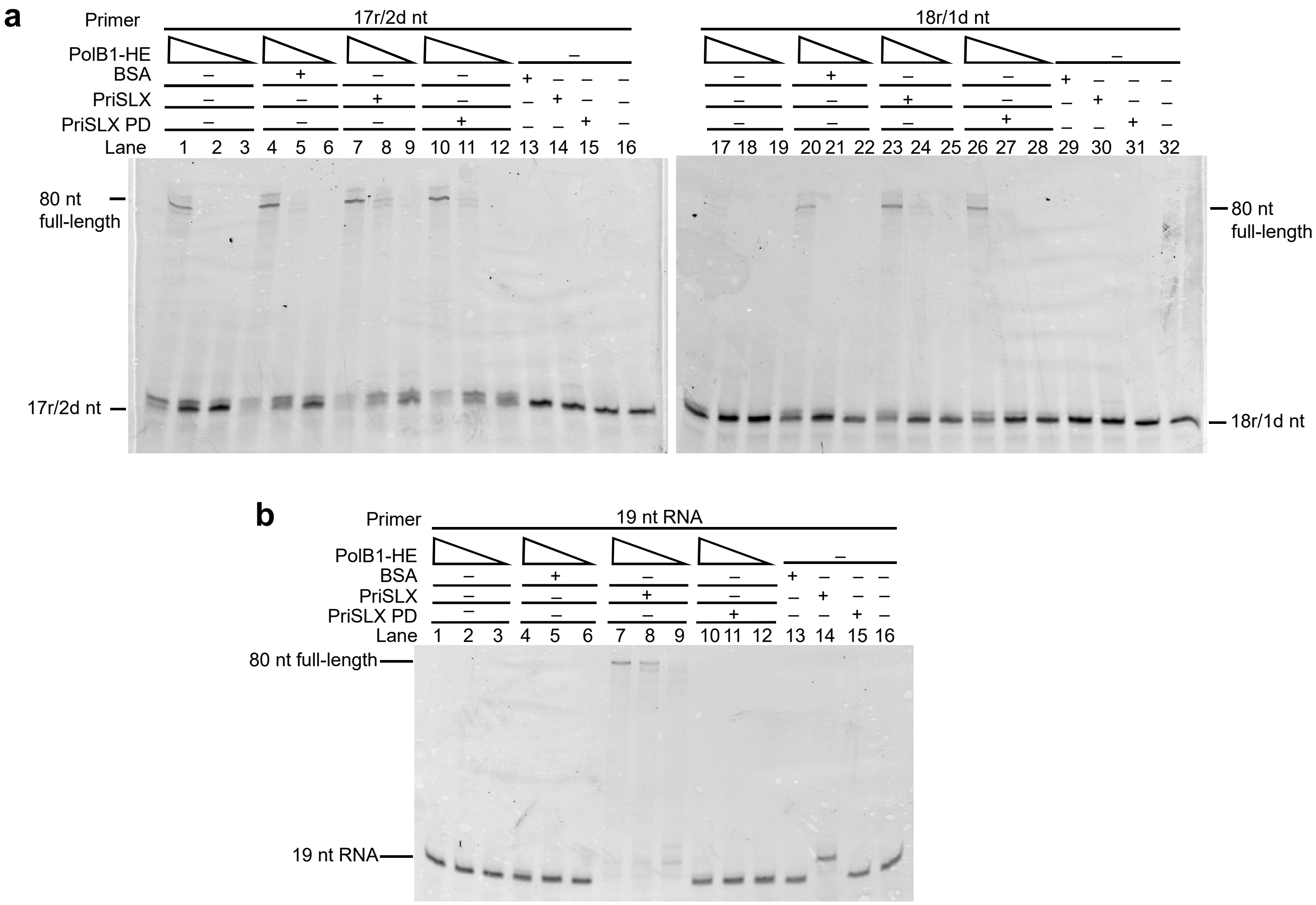

Supplementary Figure 2: Non-specific effect on hybrid-primer elongation

(a,b) Primer elongation reactions of 100 μM dNTPs; 25 nM 5'-cy5-labeled primer as indicated annealed to 80 nt template; PolB1-HE titration (25, 12.5, 6.25 nM) as indicated; 25 nM BSA as indicated; 25 nM PriSLX as indicated; and 25 nM PriSLX PD (Polymerase Dead) as indicated. Reactions were incubated for 5 minutes at 75 °C prior to termination and electrophoresis on denaturing polyacrylamide gels. 3 independent replicates.

a

	pmoles per 9 x 10^9 cells		
	dNTP	NTP	Total
(d)CTP	6.5	157	163.5
(d)TTP	8.1	215	223.1
(d)ATP	4.7	1266.9	1271.6
(d)GTP	7.1	406.7	413.8

	percentages per 9 x 10^9 cells		
	dNTP	NTP	Total
(d)CTP	3.98	96.02	100
(d)TTP	3.63	96.37	100
(d)ATP	0.37	99.63	100
(d)GTP	1.72	98.28	100
Unweighted mean	***2.42***	***97.58***	

b

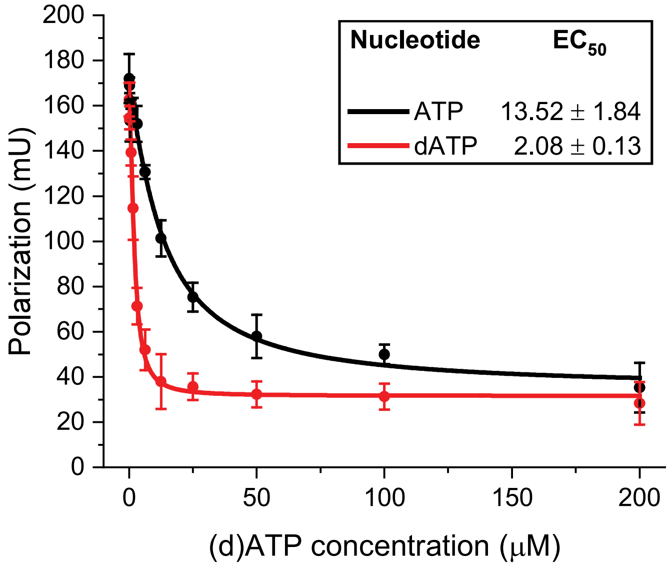

Supplementary Figure 3: Relative nucleotide abundance and binding affinity

(a) Calculation of unweighted mean of dNTP/NTP per base as determined from our previously published nucleotide abundance characterization of *Saccharolobus solfataricus* culture[26].

(b) Determination of relative NTP and dNTP binding affinity to PriSLX elongation-site. Our previously published florescence anisotropy binding data of ATP and dATP competing with fluorescently-labeled ATP for binding to elongation-competent PriSL[12] was reanalyzed by fitting to the logistic function, and the EC_{50} for ATP and dATP were derived. ATP and dATP are taken as representative of each NTP and dNTP base-nucleotide, respectively. Data points are the mean and error bars represent standard deviation.

Table 1: Oligonucleotides used in this study

Oligo	Sequence
19 nt RNA-primer	5'- /5Cy5/rCrArGrGrUrGrGrGrCrUrGrCrGrGrUrCrArCrG -3'
19 nt DNA-primer	5'- /5Cy5/CAGGTGGGCTGCGGTCACG -3'
17r/2d nt hybrid-primer	5'- /5Cy5/rCrArGrGrUrGrGrGrCrUrGrCrGrGrUrCrACG -3'
18r/1d nt hybrid-primer	5'- /5Cy5/rCrArGrGrUrGrGrGrCrUrGrCrGrGrUrCrArCG -3'
80 nt template for bulk elongation and binding assays	5'-TTAAAGTTAGGTGGCGGACTCTGCCTCAAATCGTGTAATGATGCCATGCGA CCTCTGACAACGTGACCGCAGCCCACCTG -3'
Template for RQF assays	5'-TTAAAGTTAGGTGGCGGACTCTGCCTCAAATCGTGTAATGATGCCATGCG ACCTCTGACACCGTGACCGCAGCCCACCTG -3'
5'-triphosphate 8 nt RNA-primer	5'-[ppp]rGrUrCrCrGrCrUrG-3'
poly-(24 nt T)-track template	5'- TTTTTTTTTTTTTTTTTTTTTTTTCAGCGGACACAACCAACAAACCAACA -3'

PolB1 mutagenesis	5’- [phos] AGAAGGTTTAACTGTATTATACGGTGCTACTGCTTCTTTATTCCTCC TTAATCCTCCC -3’
